# Safety and Efficacy of Photocatalytic Micro-Mist Desktop Humidifier for Dry Eye Caused by Digital Environment: A Randomized Controlled Trial

**DOI:** 10.3390/jcm13133720

**Published:** 2024-06-26

**Authors:** Reiko Arita, Shima Fukuoka

**Affiliations:** 1 Lid and Meibomian Gland Working Group (LIME), 626-11 Minami-Nakano, Minumaku, Saitama 337-0042, Japan; fshima3271@gmail.com; 2 Department of Ophthalmology, Itoh Clinic, Saitama 337-0042, Japan; 3 Omiya Hamada Eye Clinic, Saitama 330-0854, Japan

**Keywords:** dry eye, meibomian gland dysfunction, VDT work, humidifier, tear film, photocatalytic

## Abstract

**Background/Objectives**: Modern life is inconceivable without visual display terminal (VDT) work, including smartphones, computers, and games for both children and adults. VDT work under air conditioning and low humidity poses a high risk of dry eye and digital eye strain. **Methods**: Thirty-one participants were randomly divided into two groups using a desktop humidifier with photocatalytic technology, namely the “with mist” (humidifier) group and “without mist” (control) group. Participants performed VDT tasks using the humidifier with or without mist for 1 h. Ocular subjective symptoms and objective tear film parameters were assessed before, immediately after, and 1.5 h after the VDT task with or without mist. (Registry ID: UMIN000054379) **Results**: Ocular symptom scores improved significantly in the humidifier group immediately after the VDT task and up to 1.5 h later compared to before the task (*p* < 0.001, =0.006, respectively). Immediately after the VDT task, tear meniscus height was significantly higher and non-invasive breakup time was significantly longer in the humidifier group than in the control group (*p* < 0.001, =0.040, respectively). Plugging of the meibomian gland orifices was significantly reduced only in the humidifier group immediately after the VDT task compared to before the VDT task and remained significantly reduced up to 1.5 h later (*p* = 0.004, 0.016, respectively). **Conclusions**: The use of the photocatalytic desktop humidifier during VDT task resulted in significant improvements in the tear film parameters and subjective symptoms. The photocatalytic desktop humidifier could be effective in alleviating dry eye and eye strain in computer users in a modern office environment.

## 1. Introduction

According to the latest International Dry Eye Workshop Society [1], dry eye and ocular discomfort are reported to increase with Visual Display Terminal (VDT) work. Since the COVID-19 pandemic, computer use has increased dramatically, both at home and in the workplace, and the prevalence of dry eye has also increased [2]. In Japan, the proportion of households with a smartphone was around 10% in 2010, increasing to more than 80% by 2020 [3], making it a social phenomenon [3]. VDT work can cause dry eye symptoms due to increased evaporation of the tear film caused by a decrease in the number and quality of blinks [4]. Office environments with low humidity are known to exacerbate dry eye findings and symptoms [5].

The most common current treatment for alleviating dry eye symptoms in office environments is the regular use of eye drops. Wearing goggles to increase periocular humidity is also possible but presents cosmetic issues in the workplace [6].

In 2017, Wang, et al. [7] reported that the use of a USB-powered desktop humidifier that generates water vapor during VDT work increased the humidity around the eyelid area by about 5% and significantly improved subjective comfort and tear film stability, but not the aqueous and lipid layer. Subjective comfort was not evaluated on a continuous scale but was queried on a dichotomous scale.

In the present study, we used a desktop humidifier (EYE MOIST, Kaltech, Osaka, Japan) with photocatalyst technology for a higher humidification function during VDT work to examine changes in subjective comfort on a continuous scale, effects on parameters of aqueous and lipid layers of the tear film, and effects on the blinking. Here, we report the results of our study, including the persistence of these effects.

## 2. Methods

The study protocol was reviewed and approved by the Ethics Committee of the Institutional Review Board of the Yamauchi Clinic, Tokyo, Japan (Registry ID: IRIN2023-09-00202). All procedures were performed in accordance with the ethical standards of the responsible committee on human experimentation (institutional and national) and the Helsinki Declaration of 1964, as revised in 2013. Informed consent was obtained from all participants. This study was registered with the University Hospital Medical Information Network (Registry ID: UMIN000054379).

### 2.1. Participants

The study was designed as a prospective, single-center, masked trial comparing the randomized use of humidifiers with and without mist during VDT tasks performed in Itoh Clinic. A total of 31 pairs of eyes in 31 participants were enrolled. The study participants were randomly assigned by the envelope method and divided into two groups—a desktop humidifier “with mist” group (humidifier group) and “without mist” (control) group. Participants performed VDT tasks (with smartphones or handheld game consoles) while using the desktop humidifier with or without mist for 1 h. The inclusion criteria included individuals whose written consent to participate in this study had been obtained and who did not meet all the following exclusion criteria: (1) under the age of 18 or over the age of 70 when informed consent was obtained, (2) has acute ocular or eyelid disease, (3) has obvious eyelid or ocular surface disease (eyelid congenital anomalies, entropion, ectropion, Sjögren’s syndrome), (4) anti-glaucoma eye drop use, (5) has history of ocular surgery, intense pulsed light (IPL) therapy, LipiFlow^®^, or Botulinum toxin injections within 3 months, (6) shows ocular allergic symptoms at the time of examination, (7) has systemic diseases related to dry eye, (8) those on hot compresses, lid hygiene, and using eye drops or eye ointments, and (9) deemed ineligible by the principal investigator or research associate. Soft contact lenses, eye drops, eye ointment, periocular emulsion, eye cream, or eye makeup were not allowed on the day of the examination, from the morning until the end of the examination.

### 2.2. Features of Humidifier

EYE MOIST is an improved version of the beauty humidifier for the skin (KL-H01, Kaltech, Osaka, Japan) released in December 2022 for use around the eyes. It is an ultrasonic humidifier that raises the humidity around the eyes by making tap water sterile with a photocatalyst and spraying a mist of water around the eyes at a rate of 22.2 μL/s, droplet size is about 5.5 μm (Supplementary Materials File). It uses a uniquely designed spray nozzle that matches the height of each individual eye. The humidifier has a bactericidal capacity due to the arrangement of the photocatalyst [8,9] placed in the water storage area, a light to illuminate the photocatalyst, and an ultrasonic unit to atomize the water that has passed through the photocatalyst (patent number JP7428454).

### 2.3. Examinations

**Figure 1:**
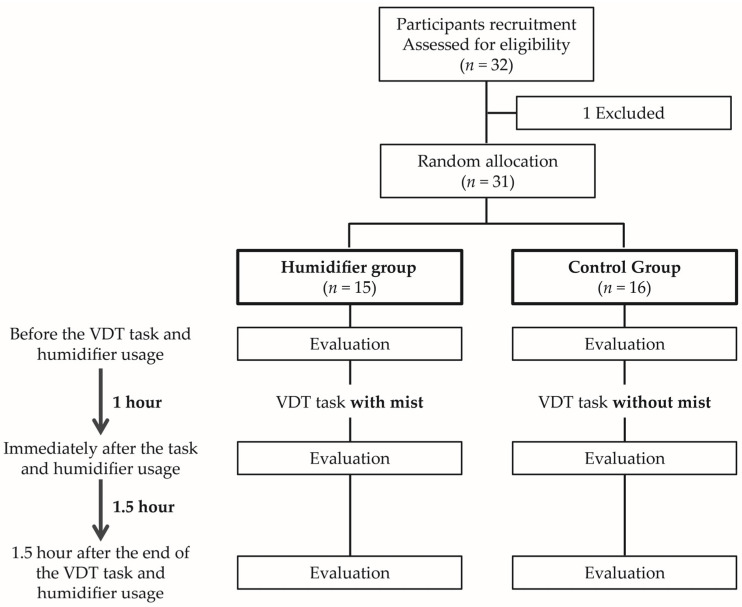
Examination procedure of the humidifier group and control group. The humidifier group and control group were interviewed and examined immediately before, immediately after, and 1.5 h after VDT task.

Ocular subjective symptoms and objective ocular surface parameters were assessed before, immediately after an hour of using the desktop humidifier with or without mist for the VDT task, and one and a half hours after the end of humidifier usage (Figure 1). The right eye was selected if both eyes were indicated. Participants were masked as to the participant assignment status and were informed in advance that the humidifier would produce “some substance” (water vapor or otherwise), which may or may not be visible depending on the environmental conditions of the room. During the break of one and a half hours following humidifier usage, participants took their time relaxing and conversing without looking at a screen or being exposed to the mist.

### 2.4. Ocular Symptoms

The ocular symptoms of each participant were assessed with the Standard Patient Evaluation of Eye Dryness (SPEED) questionnaire [10]. The visual analog scale (VAS) was used—0 (no symptoms) to 100 (maximum symptoms). An independent entry method was used for the following 7 items: dryness, eye strain, discomfort, blurred vision, foreign body sensation, eye pain, and difficulty opening eyelids.

### 2.5. Ocular Surface Parameters

Using idra (SBM Sistemi, Torino, Italy) [11,12], lipid layer thickness (LLT) (nm), blink frequency (min), complete blink number (CB), incomplete blink number (IB), tear meniscus height (TMH) (mm), and noninvasive tear film breakup time (NIBUT first, NIBUT average) (seconds) were non-invasively measured. Participants were instructed to blink naturally during the assessment of LLT and blinking. Blink frequency is the average number of blinks per minute. Incomplete blink rate (IBR) [13] was calculated by IB/(CB + IB). The number of obstructed meibomian gland orifices among the 8 orifices in the center of the upper eyelids was counted under observation with a slit lamp. Tear film breakup time with fluorescein (FBUT) (seconds) was measured three consecutive times with a stopwatch, and the mean of the three values was calculated. Fluorescein staining was used to evaluate corneal and conjunctival epithelial damage (0–9) [14]. FBUT and fluorescein staining score was examined only immediately after finishing the VDT task. As fluorescein staining could affect the measurement of LLT and NIBUT, FBUT and fluorescein scores were evaluated only immediately after the VDT task.

### 2.6. Adverse Events

Adverse events that were unfavorable to procedure were evaluated.

### 2.7. Measurement of Temperature and Relative Humidity around Humidifiers

**Figure 2:**
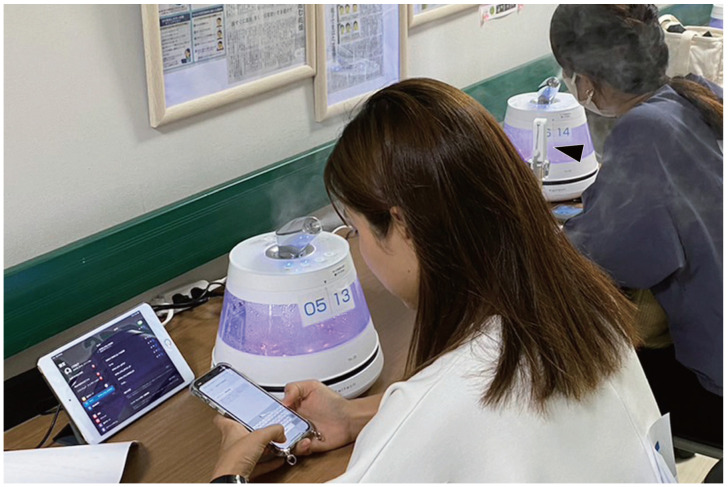
Experimental view. Participants performed VDT task using a photocatalytic humidifier with or without mist for 1 h. Temperature and relative humidity around the eyes were measured with a waterproof temperature and humidity meter (black triangle).

The temperature and relative humidity were measured locally with a waterproof temperature and humidity meter (SwitchBot Inc., Wilmington, DE, USA), situated in close proximity to the participant’s face (Figure 2).

### 2.8. Statistical Analysis

The basis of the sample size calculation was as follows: for NIBUT first, a 3.00 s difference in the mean change before and after the VDT task in the humidifier group, with a corresponding standard deviation value of 1.22 s; for NIBUT average, a 3.29 s difference in the mean change before and after the VDT task, with a corresponding standard deviation value of 2.54 s. These changes were estimated by the results of a pilot study with 12 eyes of 12 participants. Given these assumptions, we estimated that the sample size requirement would be 14 eyes in each group for a power of >90% and a significant difference at α = 0.05 with a two-sample *t*-test. With a predicted dropout rate of 10%, the required sample size was thus 31 participants. Data are shown as mean ± standard deviation (SD). The data were found to be non-normally distributed with the Shapiro–Wilk test (*p* < 0.05), and nonparametric testing was selected. The Mann–Whitney’s *U* and Fisher’s exact tests were used to compare the continuous and categorical values between the control group and the humidifier group. The Wilcoxon signed-rank test was used to compare the values before and after the VDT task. Bonferroni correction was applied for multiple comparisons. The primary and secondary end points were NIBUT first and average before and after the VDT task. All statistical analysis was performed with JMP Pro version 17 software (SAS, Cary, NC, USA). All statistical tests were two-sided, and a *p* value of <0.05 was considered statistically significant.

## 3. Results

### 3.1. Demographic Data of Participants

A total of 32 participants were screened for the study. One participant could not attend the day of the examination. A total of 31 pairs of eyes of 31 participants, including 5 men and 26 women, were enrolled. Baseline characteristics of the participants are shown in Table 1. The mean age ± SD was 41.1 ± 8.8 years (range of 21–62 years). They were randomly divided into 15 in the humidifier group and 16 in the control group. Age, sex, and medical history of the examinees are as follows, and there are no significant differences between the humidifier group and the control group (Table 1).

### 3.2. Ocular Symptoms

Baseline SPEED and VAS scores in the control group were not significantly different from those in the humidifier group (Table 2). The SPEED scores improved significantly in the humidifier group immediately after the VDT task (*p* < 0.001) (Table 2) and up to 1.5 h later (*p* = 0.006) (Table 2), compared to before the task. In the control group, there were no significant changes before and after the task (*p* = 0.059, 0.71, respectively) (Table 2). The SPEED score immediately after the VDT task was significantly less in the humidifier group than in the control group (*p* < 0.001) (Table 2). All VAS scores improved significantly in the humidifier group immediately after the task compared to before the task (Table 2). The VAS scores, except for dryness, eye pain, and difficulty opening eyelids, improved significantly up to 1.5 h after the end of task (Table 2). The humidifier group was significantly better than the control group for dryness, eye strain, foreign body sensation, and eye pain up to 1.5 h later (*p* = 0.012, 0.007, 0.005, 0.004, 0.032, respectively) (Table 2).

### 3.3. Ocular Surface Parameters

Baseline objective ocular surface parameters were not significantly different between the two groups (Table 3 and Table 4). LLT significantly increased in the humidifier group immediately after the VDT task compared to before humidifier use (*p* = 0.012) (Table 3). On the other hand, it significantly decreased in the control group immediately after the VDT task and remained significantly decreased up to 1.5 h later (*p* = 0.029, 0.013, respectively) (Table 3). Up to 1.5 h after the end of mist, the humidifier group had significantly more LLT than the control group (*p* = 0.034) (Table 3). Blink frequency and IBR did not change significantly in the two groups (Table 3). TMH significantly increased after the task compared to before in the humidifier group and significantly decreased in the control group (*p* = 0.001, 0.007, respectively). TMH immediately after the VDT task was significantly higher in the humidifier group than in the control group (*p* < 0.001) (Table 3). NIBUT first was significantly prolonged immediately after the VDT task in the humidifier group compared to the control group (*p* = 0.005) (Table 3). NIBUT first was significantly shorter in the control group 1.5 h after the end of mist than before the VDT task (*p* = 0.042) (Table 3). NIBUT first was significantly prolonged in the humidifier group immediately after the VDT task and up to 1.5 h after the end of mist compared to the control group (*p* = 0.001, 0.025, respectively) (Table 3). NIBUT average was significantly improved in the humidifier group only immediately after the VDT task (*p* = 0.017) (Table 3). NIBUT average was significantly longer in the humidifier group compared to the control group immediately after the VDT task (*p* = 0.040) (Table 3). Plugging of the meibomian gland orifices was significantly reduced only in the humidifier group immediately after the VDT task compared to before the VDT task and remained significantly reduced up to 1.5 h later (*p* = 0.004, 0.016, respectively) (Table 3). No significant change in plugging was observed in the control group (Table 3). The number of plugging immediately after the VDT task was significantly lower in the humidifier group compared to the control group and remained up to 1.5 h later (*p* < 0.001, =0.001, respectively) (Table 3). FBUT was significantly longer in the humidifier group than in the control group immediately after the VDT task (*p* < 0.001) (Table 4). Fluo scores were not significantly different between the two groups immediately after the VDT task (*p* > 0.05, respectively) (Table 4).

### 3.4. Temperature and Relative Humidity around Humidifiers

There were no significant differences in the temperature and relative humidity around the humidifiers before the experiment between the two groups (Table 5). Relative humidity significantly increased, while temperature significantly decreased immediately after the end of the VDT task in the humidifier group (*p* = 0.002, respectively), and there was no significant difference in the temperature and relative humidity 1.5 h after the VDT task compared to before the experiment (Table 5). There were no significant changes in temperature and humidity in the control group (Table 5). The mean change in relative humidity with the desktop humidifier was 22.6 ± 3.5% with the mist and −1.5 ± 2.8% without the mist. The mean difference and standard error in relative humidity change was 24.1 ± 1.3% between the two groups.

### 3.5. Adverse Events

Adverse events were not observed during and after the procedure.

## 4. Discussion

Due to continuous computer use and low relative humidity, modern humans are increasingly at risk of dry eye [1]. We found that using a photocatalytic micro-mist desktop humidifier significantly improved dry eye-related symptoms and tear film-related parameters before and after the VDT task, and that observed symptoms and other findings due to the humidifier effects persisted for up to at least 1.5 h after the end of mist. It was also found that 1 h of the VDT task without a humidifier significantly worsened dry eye-related ocular symptoms and the quantity and quality of the tear film. The results suggest that the photocatalytic micro-mist humidifier can be effective in treating digital eye strain and dry eye related to VDT, which are expected to continue increasing in the future, and is a promising treatment option other than conventional eye drop therapy, warm compresses, and blinking exercise, which may interrupt the VDT task.

It is believed that a decrease in humidity increases the amount and rate of evaporation of the tear film and reduces tear film stability, resulting in ocular discomfort [1,15]. In this study, the relative humidity around the desktop humidifier significantly increased immediately after mist spraying compared to before mist spraying in the humidifier group but did not change in the control group. LLT increased significantly immediately after mist spraying in the humidifier group. The number of plugging was significantly decreased in the humidifier group compared to the control group and the efficacy continued up to 1.5 h after the end of mist. In the previous report, the humidity change was +3.1 ± 3.1% for the humidifier group and −2.3 ± 4.4% for the control group, with a humidity difference of +5.4 ± 5.0% [7], while in our study the mean ± SD of humidity change was +22.6 ± 3.5% for the humidifier group and −1.5 ± 2.8% for the control group, with a difference was 24.1 ± 1.3% (mean ± SE) between the two groups. No paper has investigated whether a single ophthalmic solution reduces plugging of the meibomian glands. There is a paper that showed an increase in the lipid layer thickness with a single diquafosol ophthalmic solution [16], but not with an artificial tear solution. Artificial tear solutions on the eyelid margin evaporate soon after the eye drop is applied and is not effective enough to reduce plugging of the eyelid margin. However, the photocatalytic micro-mist tabletop humidifier used in this study increased the humidity around the eyelid to approximately 70% (relative humidity change of 22.6%) and maintained high humidity for 1 h, which may have deflated the oil at the orifices of meibomian glands and reduced plugging. The desktop humidifier used in the previous study [7] increased the humidity around the eyelid by only about 5.4%. This was the first study to suggest that continuous exposure to mist (i.e., maintaining high humidity in the eyelid area) was effective in reducing plugging of the meibomian glands. These results suggested that when plugging was exposed to micro-mist generated by the humidifier for 1 h, this reduced the blistering and orifice clogging, resulting in a significant improvement in LLT. In this study, we chose to non-invasively measure the number of plugging and LLT rather than perform an invasive assessment such as meibomian gland expressibility or meibum quality. Invasive tests can affect the results of subsequent tear film examinations. Further studies at different relative humidity with different rates of humidifier mist are needed to determine the optimum relative humidity around the eyes for reducing plugging and increasing LLT. TMH was significantly increased only in the humidifier group. The increase in TMH in the humidifier group was due to the increase in periocular relative humidity caused by the micro-mist generated by the humidifier, while the decrease in TMH in the control group was due to the continuation of the VDT task under relatively low humidity conditions. Immediately after 1 h of VDT task, NIBUT first was prolonged by 3.37 s, and even up to 1.5 h later by 1.85 s on average. The possible reason for this was thought to be an improvement in both the lipid and aqueous layers of the tear film. The mechanism was speculated to be due to the increased relative humidity caused by the mist exposure, which increased the tear meniscus, soaked the obstruction of the meibomian gland orifice, melted the lipids, and improved the stability of the tear film.

After the VDT task, both the aqueous and lipid layers were significantly worse in the absence of mist in the control group. The thinning of the lipid layer of the tear film was found to persist for at least up to 1.5 h after the mist was completed. The reason why dry eye-related symptoms and digital eyestrain symptoms are more intense in the evening or later may be that the tear film condition does not easily return to its original state in the low-humidity environment of an office [17,18]. Moreover, dry eye have also been reported to reduce work productivity and concentration in office workers [19].

The reason why there was no change in the number of blinks in both groups may be that the blink measurements with idra were taken before and after the VDT task, not during the task. We also wanted to examine the effect of the humidifier on tear fluid parameters before and after VDT task, so we used idra in this case. The measurement of the blink itself should be performed during the VDT task, and future studies will measure the number of blinks during the task.

In participants using the humidifier with mist, all the VAS items significantly improved immediately after the VDT task, and the effects on some symptoms (eye strain, discomfort, blurred vision, and foreign body sensation) persisted for up to 1.5 h after the end of mist spraying ended. This is presumably because the relative humidity increased to over 70% and the micro-mist was evenly distributed around the eyes, creating a comfortable environment. This is consistent with previous reports of changes in relative humidity affecting the symptoms of dry eye symptoms [1,7,20].

Wang, et al. [7] found significant prolongation of NIBUT and improvement in subjective symptoms before and after 1 h of VDT work while using a USB-powered desktop humidifier. Tear meniscus and lipid layer grades were not significantly different from controls. In this study, not only NIBUT but also TMH and lipid layer parameters were significantly improved. The differences in the results seemed to be because of differences in the function of the humidifiers. Hirayama, et al. [20] reported the effectiveness of the Moist Cool Air Device Electrospray Apparatus (MCAD) on office workers performing around 4 h of VDT work. The device delivered positively charged droplets to the ocular surface. Subjective symptoms, functional visual acuity, FBUT, strip meniscometry, and tear film evaporation rate showed significant improvement, while lipid layer stability and corneal staining score showed no significant change. The difference between MCAD and EYE MOIST is that MCAD has a spray volume of 0.3 μL/s, a droplet size of 100 μm and has safety concerns regarding the possible exposure and inhalation of castor oil particles, whereas EYE MOIST has a spray volume of 22.2 μL/s, a droplet size of 5.5 μm and uses photocatalytic water that is sterilized and safe.

This study was a randomized controlled trial and was masked as to participant assignment status. Participants were informed in advance that the humidifier would produce “some substance”, which may or may not be visible. Although it is difficult to mask the generation of water vapor, the participants were also informed of the possibility of “some invisible component” being generated from the humidifier even without mist, so we believe that there was little bias in the collection of symptoms. Eighty percent of the participants in this study were women. Tears are affected by sex hormones, but since EYEMOIST is a mechanism that physically moistens the periocular area by spraying micro-mist around the eye, there seems to be no gender difference in its effect. However, we would like to increase the number of male participants in the future to verify whether there is a difference in effectiveness between men and women. Although the present follow-up was up to 1.5 h after the end of mist, a longer follow-up is desirable. The participants in this study included dry eye patients, but they also included people predisposed to dry eye and normal eyes. Some participants originally complained of eyestrain, while others had no eyestrain. This experiment may be applicable to all VDT workers. In the future, we would like to examine the usefulness of this desktop humidifier for a wider range of age groups and for participants who perform VDT work for longer periods of time.

## 5. Conclusions

The use of the EYE MOIST photocatalytic desktop humidifier during the VDT tasks resulted in significant improvements in the tear film and meibomian gland parameters and subjective symptoms such as eye strain and eye dryness. The photocatalytic desktop humidifier could be effective in alleviating dry eye and eye strain in computer users in a modern office environment.

## Figures and Tables

**Table 1:** Baseline characteristics of the humidifier and the control groups.

Characteristic	All (*n* = 31)	Humidifier Group (*n* = 15)	Control Group (*n* = 16)	*p* Value
Age, mean ± SD (range) (years)	41.1 ± 8.8 (21–62)	41.7 ± 9.2 (25–62)	40.5 ± 8.7 (21–60)	0.86
Sex (male/female)	5/26	2/13	3/13	1.0
History of dry eye (*n* (%))	14 (45%)	6 (40%)	8 (50%)	0.72
Contact lens wear (*n* (%))	4 (13%)	3 (20%)	1 (6%)	0.33
Allergic conjunctivitis (*n* (%))	2 (6%)	2 (13%)	0 (0%)	0.23
Seasonal allergy (*n* (%))	12 (39%)	8 (53%)	4 (25%)	0.15

*p* values were obtained with Mann–Whitney’s *U* test or Fisher’s exact test.

**Table 2:** Time course of subjective symptoms before, immediately after, and 1.5 h after visual display terminal task of the humidifier and the control groups.

Symptom		Humidifier Group (*n* = 15)	*p* Value vs. Before	Control Group (*n* =16)	*p* Value vs. Before	*p* Value for Humidifier vs. Control
SPEED (0–28)	Before	8.3 ± 4.6		9.3 ± 5.0		0.61
	Immediately after	2.0 ± 2.6	<0.001 **	7.1 ± 4.3	0.059	<0.001 ††
	1.5 h after	6.0 ± 4.5	0.006 *	8.3 ± 5.2	0.71	0.22
VAS score (0–100)						
Dryness	Before	41.5 ± 27.6		55.0 ± 29.0		0.17
	Immediately after	9.9 ± 14.2	0.002 *	49.4 ± 34.2	1.0	0.001 †
	1.5 h after	32.3 ± 24.6	0.47	58.4 ± 26.6	0.51	0.012 †
Eye strain	Before	49.9 ± 23.9		58.9 ± 29.2		0.28
	Immediately after	14.7 ± 16.6	<0.001 **	51.4 ± 34.6	1.0	0.003 †
	1.5 h after	28.4 ± 24.4	0.004 *	58.6 ± 28.2	1.0	0.007 †
Discomfort	Before	32.0 ± 23.3		44.4 ± 29.5		0.25
	Immediately after	7.7 ± 15.9	0.002 *	39.1 ± 35.2	0.88	0.002 †
	1.5 h after	13.1 ± 20.2	0.035 *	43.5 ± 30.6	1.0	0.005 †
Blurred vision	Before	38.5 ± 24.1		38.6 ± 33.8		0.87
	Immediately after	9.7 ± 17.0	<0.001 **	29.3 ± 28.1	0.25	0.029 †
	1.5 h after	15.9 ± 18.4	<0.001 **	32.6 ± 31.7	0.070	0.23
Foreign body sensation	Before	28.9 ± 26.6		37.3 ± 28.4		0.47
	Immediately after	3.3 ± 6.2	0.002 *	34.5 ± 29.6	1.0	<0.001 ††
	1.5 h after	4.5 ± 9.4	0.002 *	34.5 ± 34.0	1.0	0.004 †
Eye pain	Before	17.3 ± 19.5		21.4 ± 17.8		0.51
	Immediately after	2.3 ± 5.0	0.004 *	18.8 ± 17.7	1.0	0.005 †
	1.5 h after	6.1 ± 12.9	0.098	23.8 ± 25.3	1.0	0.032 †
Difficulty opening eyelids	Before	21.4 ± 30.9		21.6 ± 20.8		0.60
	Immediately after	3.7 ± 5.5	0.016 *	24.4 ± 27.6	1.0	0.031 †
	1.5 h after	12.8 ± 19.4	0.59	32.3 ± 32.2	0.082	0.074

Data are mean ± SD. * Adjusted *p* < 0.05 and ** Adjusted *p* < 0.001 versus corresponding value before visual display terminal task (the Wilcoxon signed-rank test with Bonferroni correction for two comparisons). † *p* < 0.05 and †† *p* < 0.001 (the Mann–Whitney’s *U* test). SPEED, Standardized Patient Evaluation of Eye Dryness; VAS, Visual Analogue Scale.

**Table 3:** Time course of ocular parameters before, immediately after, and 1.5 h after visual display terminal task of the humidifier and the control groups.

Characteristic		Humidifier Group (*n* = 15)	*p* Value vs. Before	Control Group (*n* = 16)	*p* Value vs. Before	*p* Value for Humidifier vs. Control
LLT (nm)	Before	68.5 ± 10.9		75.5 ± 21.2		0.50
	Immediately after	79.2 ± 13.0	0.012 *	66.6 ± 16.6	0.029 *	0.05
	1.5 h after	67.9 ± 8.5	1.0	59.4 ± 15.2	0.013 *	0.034 †
Blink frequency (/min)	Before	32.2 ± 18.9		39.0 ± 26.7		0.74
	Immediately after	24.9 ± 9.9	0.17	28.9 ± 22.3	0.15	0.97
	1.5 h after	27.9 ± 14.6	1.0	24.1 ± 10.8	0.17	0.43
IBR	Before	0.3 ± 0.3		0.4 ± 0.3		0.19
	Immediately after	0.4 ± 0.3	0.83	0.2 ± 0.2	0.31	0.078
	1.5 h after	0.4 ± 0.4	0.59	0.4 ± 0.4	1.0	0.89
TMH (mm)	Before	0.24 ± 0.09		0.26 ± 0.08		0.42
	Immediately after	0.32 ± 0.11	0.001 *	0.20 ± 0.06	0.007 *	<0.001 ††
	1.5 h after	0.24 ± 0.06	1.0	0.21 ± 0.05	0.13	0.43
NIBUT first (s)	Before	6.28 ± 2.24		7.36 ± 2.67		0.24
	Immediately after	9.65 ± 2.55	0.005 *	6.20 ± 2.45	0.15	0.001 †
	1.5 h after	8.13 ± 3.10	0.27	5.41 ± 1.91	0.042 *	0.025 †
NIBUT average (s)	Before	8.72 ± 1.60		9.55 ± 2.57		0.21
	Immediately after	11.02 ± 2.82	0.017 *	8.78 ± 2.19	0.49	0.040 †
	1.5 h after	9.61 ± 2.47	0.65	8.16 ± 2.47	0.32	0.23
Number of obstructed meibomian gland orifices (0–8)	Before	3.0 ± 2.7		4.6 ± 2.1		0.057
	Immediately after	1.2 ± 1.5	0.004 *	4.6 ± 2.1	1.0	<0.001 ††
	1.5 h after	1.7 ± 1.9	0.016 *	4.6 ± 2.1	1.0	0.001 †

Data are mean ± SD. * Adjusted *p* < 0.05 versus corresponding value before visual display terminal task (the Wilcoxon signed-rank test with Bonferroni correction for two comparisons). † *p* < 0.05 and †† *p* < 0.001 (the Mann–Whitney’s *U* test). LLT, lipid layer thickness; IBR, incomplete blink rate; TMH, tear meniscus height; NIBUT, noninvasive breakup time of the tear film.

**Table 4:** Comparison of tear film breakup time with fluorescein staining immediately after visual display terminal task of the humidifier and the control groups.

Characteristic	Humidifier Group (*n* = 15)	Control Group (*n* = 16)	*p* Value
FBUT (s)	6.8 ± 2.4	3.2 ± 1.2	<0.001 **
Corneal fluo score (0–3)	0.0 ± 0.0	0.3 ± 0.7	0.18
Temporal conjunctival fluo score (0–3)	0.4 ± 0.8	0.4 ± 0.9	1.0
Nasal conjunctival fluo score (0–3)	0.6 ± 1.0	0.4 ± 0.9	0.62
Corneal and conjunctival fluo score (0–9)	1.0 ± 1.7	1.1 ± 2.4	0.73

Data are mean ± SD. *p* values were obtained with the Mann–Whitney’s *U* test. ** *p* < 0.001. FBUT, tear film breakup time with fluorescein; fluo score, fluorescein staining score.

**Table 5:** Time course of temperature and relative humidity around the humidifiers before, immediately after, and 1.5 h after the visual display terminal task of the humidifier and the control groups.

Characteristic		Humidifier Group	*p* Value vs. Before	Control Group	*p* Value vs. Before	*p* Value for Humidifier vs. Control
Temperature (°C)	Before	24.5 ± 0.6		24.4 ± 0.3		0.55
	Immediately after	22.2 ± 1.1	0.002 *	24.4 ± 0.5	1.0	0.003 †
	1.5 h after	25.1 ± 0.3	0.080	25.0 ± 0.2	0.063	0.21
Relative humidity (%)	Before	47.6 ± 1.9		47.0 ± 0.9		0.11
	Immediately after	70.3 ± 2.6	0.002 *	45.5 ± 2.4	0.19	<0.001 ††
	1.5 h after	44.5 ± 2.5	0.051	45.4 ± 1.7	0.070	0.55

Data are mean ± SD. * Adjusted *p* < 0.05 versus corresponding value before visual display terminal task (the Wilcoxon signed-rank test with Bonferroni correction for two comparisons). † *p* < 0.05 and †† *p* < 0.001 (the Mann–Whitney’s *U* test).

## Data Availability

Authors can share our research data, if requested.

## Supplementary Materials

The following supporting information can be downloaded at: https://www.mdpi.com/article/10.3390/jcm13133720/s1.
