# Supplementary material for: Safety and Efficacy of Photocatalytic Micro-Mist Desktop Humidifier for Dry Eye Caused by Digital Environment: A Randomized Controlled Trial
Source: J Clin Med. 2024 Jun 26;13(13):3720. doi: 10.3390/jcm13133720 (PMC11242111; doi:10.3390/jcm13133720)
Supplement: Supplementary file 1 [file jcm-13-03720-s001.zip › EYE MOIST Micro Mist Patent.pdf]

[Regarding patents]
Patent number JP7428454
Date of registration: January 29, 2024
Application number: Patent application 2023-512681
Application date: December 22, 2022
Name of invention: "Humidifier"
Patent holder: Kaltech Co., Ltd.

Summary of the Invention
"A photocatalyst is placed in the area where the water is stored, and a light shines on the photocatalyst.
By arranging an ultrasonic unit to atomize the water that has passed through the photocatalyst, sterilizing capabilities.

[Mist-related technology]
"There are multiple water mist ejection devices prepared,
At least one of the blowing members has a magnet or a tag attached thereto;
Further comprising a sensor for detecting the magnet or tag;
When the sensor detects the magnet or tag,
The control unit, according to a control program corresponding to the blowing member,
At least control the fan.

[Information regarding comparison with existing research]

1. 80ml/h = 1.33 ml/min = 1330 μl/min = 22.2 μl/sec

2. Droplet size
   The size of the mist
   The prototype used in the clinical trials in September was 5.5μ
   EYE MOIST is 3.5μ

3. Noise level
   The prototype used in the clinical trials in September was 40dB.
   EYE MOIST is 30dB
