# Supplementary material for: Safety and Efficacy of Photocatalytic Micro-Mist Desktop Humidifier for Dry Eye Caused by Digital Environment: A Randomized Controlled Trial
Source: J Clin Med. 2024 Jun 26;13(13):3720. doi: 10.3390/jcm13133720 (PMC11242111; doi:10.3390/jcm13133720)
Supplement: Supplementary file 1 [file jcm-13-03720-s001.zip › MIST-SIZE.pdf]

Atomized particle diameter

Test Report

Report number: Lab-TR-202304001-1

佛山市南海科日超声电子有限公司 检测中心 检验报告专用章

Experimental application form: KR-TR-202304001   Sample quantity: 2 PCS   Testing time: 2023-04-04 — 2023-04-04

Detection object<br>MU455	Rated working voltage(V)	AC 100-240	Machine power (W)	/
	Atomizer characteristics	Diameter (mm): 25 Resonant frequency (MHz): 1.7 Coating: Glass glaze Manufacturer/Batch No.: Keri		
Purpose of testing	Diameter of atomized particles after the test sample was replaced with a new nozzle			
testing base	C1-28 Atomized Particle Diameter Test Operation Instructions (A0)			
Test conditions	Room temperature(ÿ)	Start test: 24.3 End test: 24.5	Relative humidity (%)	Start test: 58.4 End test: 60.2
Modify information	The nozzle of the prototype was replaced, the atomization was fixed at H gear, and the wind speed PWM value was 30%.			
Test results				

Test items / Detection gear	D10 (μm)	D50 (μm)	D90 (μm)
1#	5.48	6.66	7.99
2#	5.56	6.64	7.91

Instrument name and number	Laser particle size analyzer (DYQ130352)
in conclusion	Data collection, no judgment.
picture	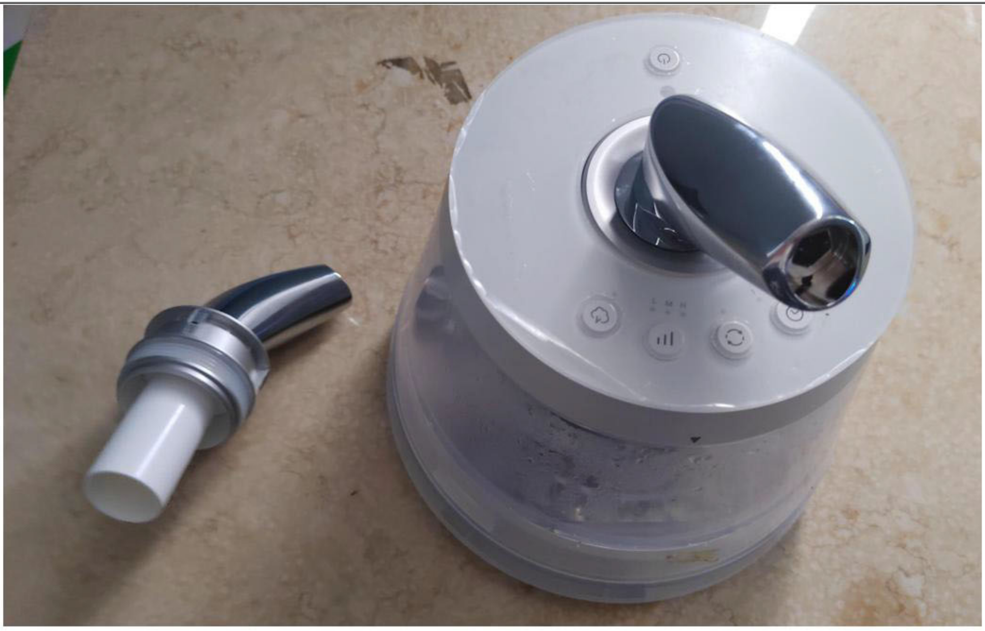 Test samples

prepared by: 王婉颖

approve: 蔡汝昌

OMEC Instruments

Particle size test report

Instrument Type DP-02 average

Sample name: MU455	**Sample refractive index: 2.60**	**Analysis mode: polydis.**
Sample number: 1#	**Dispersion medium: water**	**Fitted residual: 0.42**
Ultrasonic time: 15s	**Medium refractive index: 1.33**	**Shading ratio: 70.9%**
Test date: 2023/4/4	**Dispersant: Glycerol**	**Cutoff lower limit: 1.00**
Test time: 11:20:58	**Dispersant dosage: 1**	**Cutoff limit: 1500.00**

Particle size characteristic parameters

D(4,3)	6.67 μm	D50	6.66 μm	D(3,2)	6.54 μm	SSA	0.92 sq.m/cc
D10	5.48 μm	D25	6.02 μm	D75	7.26 μm	D90	7.99 μm

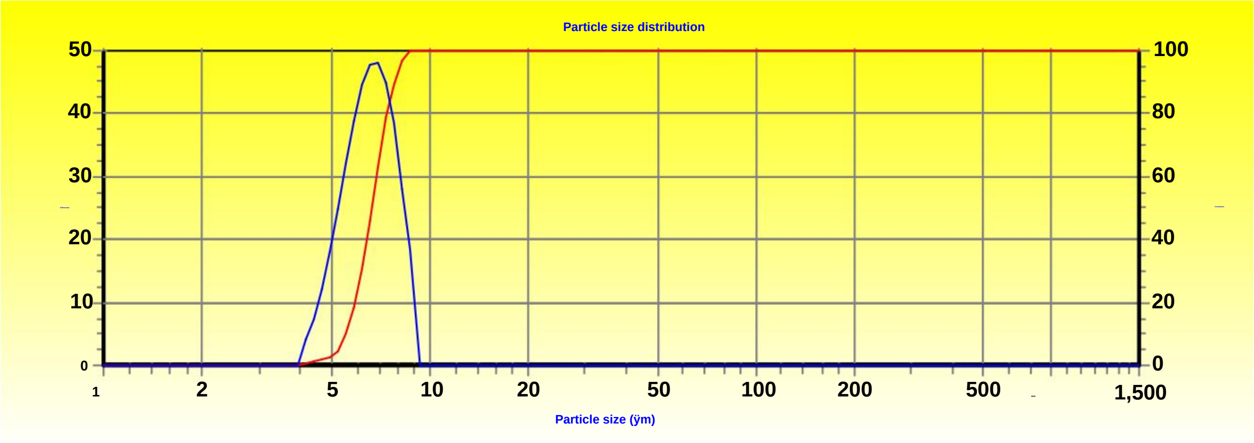

Particle size distribution table

Particle size (μm)	Differential distribution (%)	Cumulative distribution (%)	Particle size (μm)	Differential distribution (%)	Cumulative distribution (%) (%)	Particle size (μm)	Differential distribution	Cumulative distribution (%)
1.00			12.15	0.00	100.00	147.58	0.00	100.00
1.20	0.00	0.00	14.52	0.00	100.00	176.40	0.00	100.00
1.43	0.00	0.00	17.36	0.00	100.00	210.85	0.00	100.00
1.71	0.00	0.00	20.75	0.00	100.00	252.02	0.00	100.00
2.04	0.00	0.00	24.80	0.00	100.00	301.23	0.00	100.00
2.44	0.00	0.00	29.64	0.00	100.00	360.05	0.00	100.00
2.92	0.00	0.00	35.43	0.00	100.00	430.36	0.00	100.00
3.49	0.00	0.00	42.34	0.00	100.00	514.40	0.00	100.00
4.17	0.62	0.62	50.61	0.00	100.00	614.84	0.00	100.00
4.98	3.31	3.92	60.49	0.00	100.00	734.90	0.00	100.00
5.95	19.23	23.15	72.31	0.00	100.00	878.40	0.00	100.00
7.11	46.02	69.17	86.43	0.00	100.00	1049.93	0.00	100.00
8.50	29.49	98.66	103.30	0.00	100.00	1254.95	0.00	100.00
10.16	1.34	100.00	123.47	0.00	100.00	1500.00	0.00	100.00

Approval: 蔡汝昌 **Detection:** 王婉颖

Device number: 331110003
Injection system: Cycle
Measurement parameters: current.mdp2
file name:

OMEC Instruments

Particle size test report

Instrument Type DP-02 average

**Sample name:** MU455	**Sample refractive index: 2.60**	**Analysis mode: polydis.**
**Sample No.:** 2#	**Dispersion medium: water**	**Fitting residual: 0.40**
**Ultrasonic time: 15s**	**Medium refractive index: 1.33**	**Shading ratio: 76.0%**
**Test date: 2023/4/4**	**Dispersant: Glycerol**	**Cutoff lower limit: 1.00**
**Test time: 11:56:19**	**Dispersant dosage: 1**	**Cutoff limit: 1500.00**

Particle size characteristic parameters

D(4,3)	6.67 ÿμm	D50	6.64 μm	D(3,2)	6.57 μm	SSA	0.91 sq.m/cc
D10	5.56 μm	D25	6.05 μm	D75	7.20 μm	D90	7.91 μm

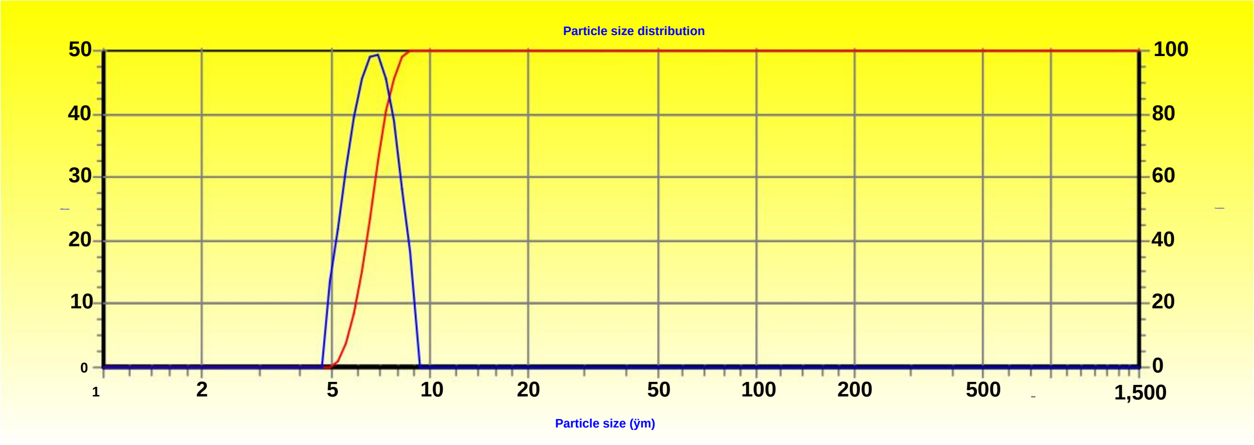

Particle size distribution table

Particle size (μm)	Differential distribution (%)	Cumulative distribution (%)	Particle size (μm)	Differential distribution (%)	Cumulative distribution (%) (%)	Particle size (μm)	Differential distribution	Cumulative distribution (%)
1.00			12.15	0.00	100.00	147.58	0.00	100.00
1.20	0.00	0.00	14.52	0.00	100.00	176.40	0.00	100.00
1.43	0.00	0.00	17.36	0.00	100.00	210.85	0.00	100.00
1.71	0.00	0.00	20.75	0.00	100.00	252.02	0.00	100.00
2.04	0.00	0.00	24.80	0.00	100.00	301.23	0.00	100.00
2.44	0.00	0.00	29.64	0.00	100.00	360.05	0.00	100.00
2.92	0.00	0.00	35.43	0.00	100.00	430.36	0.00	100.00
3.49	0.00	0.00	42.34	0.00	100.00	514.40	0.00	100.00
4.17	0.01	0.01	50.61	0.00	100.00	614.84	0.00	100.00
4.98	1.50	1.51	60.49	0.00	100.00	734.90	0.00	100.00
5.95	20.56	22.07	72.31	0.00	100.00	878.40	0.00	100.00
7.11	49.05	71.13	86.43	0.00	100.00	1049.93	0.00	100.00
8.50	28.54	99.66	103.30	0.00	100.00	1254.95	0.00	100.00
10.16	0.34	100.00	123.47	0.00	100.00	1500.00	0.00	100.00

Approval: 蔡汝昌 Detection: 王婉颖

Device number: 331110003
Injection system: Cycle
Measurement parameters: current.mdp2
file name:
