# Supplementary material for: Safety and Efficacy of Photocatalytic Micro-Mist Desktop Humidifier for Dry Eye Caused by Digital Environment: A Randomized Controlled Trial
Source: J Clin Med. 2024 Jun 26;13(13):3720. doi: 10.3390/jcm13133720 (PMC11242111; doi:10.3390/jcm13133720)
Supplement: Supplementary file 1 [file jcm-13-03720-s001.zip › spray volume .pdf]

Machine Translated by Google

Wet Desktop

Test Report

Report number: Lab-TR-202212017-11

检测中心 检验报告专用章

Experimental application form: KR-TR-202212017 Sample quantity: 1 PC Test time: 2022-12-30 — 2022-12-30

Detection object<br>MU455	Rated working voltage(V)	Input 36V power not less than 55W	Overvoltage protection (V)	ÿ37.5
	Machine power (W)<br>(with electromagnet)	Heavy fog: 44±3<br>Medium fog level: 30±3<br>Light fog: 22±3	Machine power (W)<br>(without electromagnet)	Heavy fog: 40±3<br>Medium fog level: 27±3<br>Light fog: 18±3
	Atomizer characteristics Diameter (mm): 25 Resonant frequency (MHz): 1.7 Coating: Glass glaze Manufacturer/Batch No.: Keri			
Purpose of testing	Test sample wet desktop condition			
testing base	C1-59 Humidifier Low Temperature Simulation Use Test Operation Instructions (A0), MU455 Product Specification A 2022.12.20			
Test conditions	Room temperature(ÿ)	Start test: 20.3 End test: 21.7 Relative humidity (%) Start test: 31.4 End test: 75.3		
	Detection method	1. Initial humidity in the constant temperature box: 30±2%RH, temperature: 20±2ÿ;<br>2. The prototype is placed in a 30m³ small glass room in a 100m³ walk-in constant temperature and humidity chamber, and the fog mode is turned on. The humidity probe is placed 30cm behind the prototype;<br>3. Record the water droplets on the surface of the prototype and the ground.		
other instructions	1. Main control board checksum: 0fc0;<br>2. Keyboard screen printing 2022.11.30 Checksum: ac4d;<br>3. Water inspection board checksum: 2BD3.<br>4. The parameters of this machine do not include the definition of the amount of fog, because it is related to the structural design of the whole machine. The gears defined by the customer are 70mL/h, 200mL/h, 300mL/h.			

Test results

Sample No.	Average humidification capacity (mL/h)	Test results	Individual judgment
1#	260.8	After running to 70%RH humidity, no water droplets appeared on the panel and the ground of the prototype.	qualified

Instrument name and number	Electronic scale (DYQ120144), walk-in constant temperature and humidity chamber (GYQ150132)
in conclusion	ÿ Qualified<br>ÿ Unqualified
illustrate	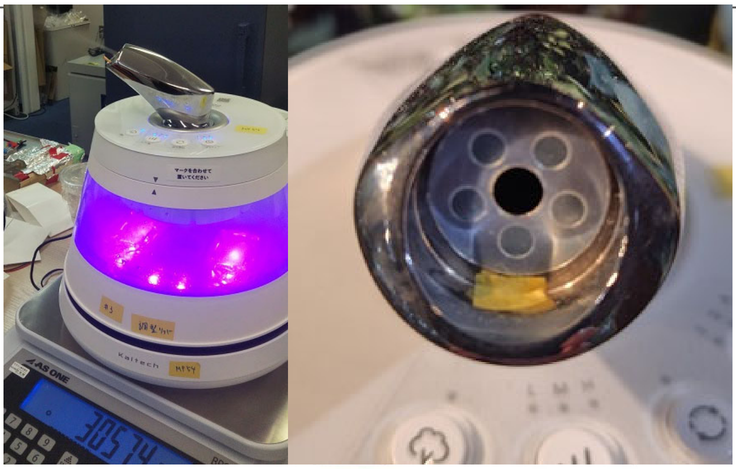<br>Wet table test

prepared by: 王婉颖 approve: 蔡汝昌

湿桌面
检测报告

报告编号: Lab-TR-20221201７-11

实验申请单：KR-TR-202212017　　样品数量：1 PC　　测试时间: 2022-12-30 -- 2022-12-30

佛山市南海科日超声电子有限公司 检测中心 检验报告专用章

**检测对象**<br>MU455	额定工作电压(V)	输入 36V 功率不低于 55W	过压保护（V）	≥37.5
	整机功率(W)<br>（带电磁铁）	大雾档：44±3<br>中雾档：30±3<br>小雾档：22±3	整机功率(W)<br>（不带电磁铁）	大雾档：40±3<br>中雾档：27±3<br>小雾档：18±3
	雾化片特性	直径(mm)：25　谐振频率(MHz)：1.7　镀层：玻璃釉　生产商/批号：科日		
**检测目的**	测试样机湿桌面情况			
**检测依据**	C1-59　加湿机低温模拟使用检测作业指导书(A0)、MU455 产品规格书 A 2022.12.20			
**检测条件**	室温(℃)	开始测试：20.3　结束测试：21.7	相对湿度(%)	开始测试：31.4　结束测试：75.3
	检测方法	1、恒温箱内初始湿度：30±2%RH，温度：20±2℃；<br>2、样机放置于 100m³ 步入式恒温恒湿箱里的 30m³ 小玻璃房开启大雾档运行，湿度探头放置于样机后方 30cm 处；<br>3、记录样机表面及地面积水珠情况。		
**其他说明**	1、主控板校验和：0fc0；<br>2、按键板丝印 2022.11.30 校验和：ac4d；<br>3、检水板校验和：2BD3。<br>4、本整机参数不包括出雾量定义，因为与整机结构设计有关，客户自己定义的档位为 70mL/h、200mL/h、300mL/h。			
**检测结果**				
**样品编号**	**平均加湿量(mL/h)**	**检测结果**		**单项判定**
1#	260.8	运行至 70%RH 湿度后，样机面板和地面没有出现水珠。		合格
**仪器名称及编号**	电子称（DYQ120144）、步入式恒温恒湿箱（GYQ150132）			
**结论**	■ 合格<br>□ 不合格			
**图片说明**	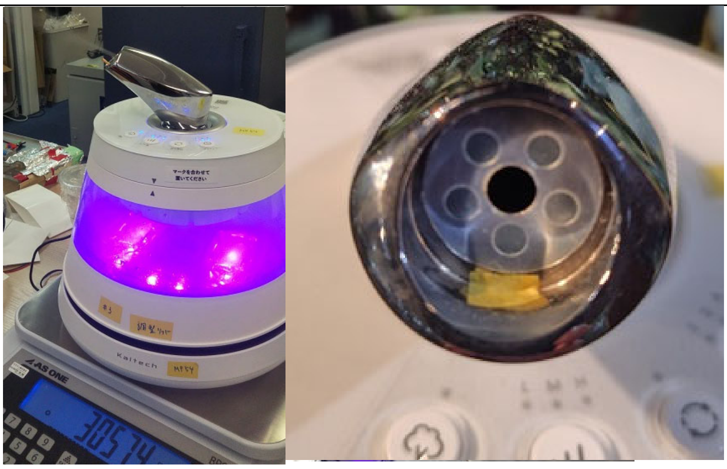<br>湿桌面测试			

编制:王婉颖　　　　批准:蔡汝昌

EYE MOIST (T3) Spray volume measurement	Measurement date: May 17, 2023	Measurement location:

Medium Mode	Spray time	0 h	1h		2h	
	Temperature/humidity	25.8ÿ/31%	25.4ÿ/49%		25.7ÿ/45%	
		weight (g)	weight (g)	Spray volume (g)	weight (g)	Spray volume (g)
Sample No.	P1	643.5	566.5	77.0	482.5	84.0
	P3	643.5	579.0	64.5	502.0	77.0
	P4	643.5	578.5	65.0	503.0	75.5
	P5	643.5	570.5	73.0	494.0	76.5
	S1	643.5	569.5	74.0	490.5	79.0
	S2	643.0	579.0	64.0	504.5	74.5
	S3	643.0	568.5	74.5	486.0	82.5
	S4	644.0	578.0	66.0	503.0	75.0
	S5	644.0	569.0	75.0	491.0	78.0
	G1	644.0	575.5	68.5	498.0	77.5
	G2	644.0	577.0	67.0	505.5	71.5
	G3	644.0	570.5	73.5	490.0	80.5
	G4	643.0	575.5	67.5	500.0	75.5
	G5	644.0	577.5	66.5	502.0	75.5
	C6	645.5	576.5	69.0	502.0	74.5
	C7	645.0	573.5	71.5	499.0	74.5
	C8	645.5	580.0	65.5	507.0	73.0

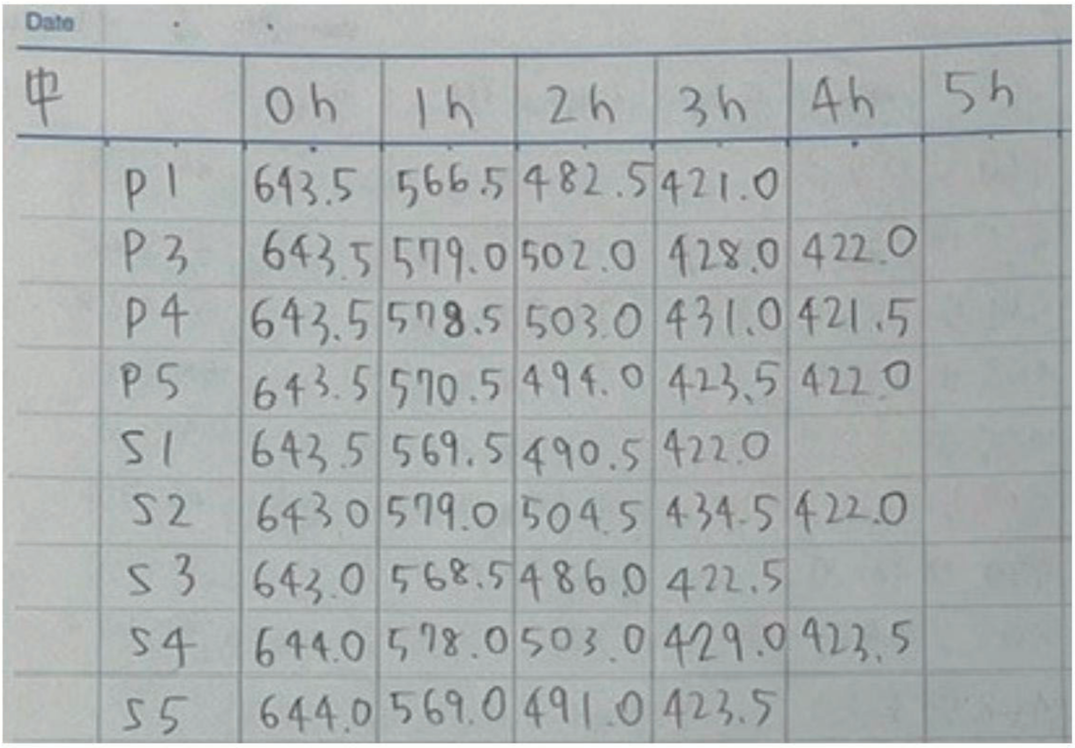
Date

中		0h	1h	2h	3h	4h	5h
	P1	643.5	566.5	482.5	421.0		
	P3	643.5	579.0	502.0	428.0	422.0	
	P4	643.5	578.5	503.0	431.0	421.5	
	P5	643.5	570.5	494.0	423.5	422.0	
	S1	643.5	569.5	490.5	422.0		
	S2	643.0	579.0	504.5	434.5	422.0	
	S3	643.0	568.5	486.0	422.5		
	S4	644.0	578.0	503.0	429.0	423.5	
	S5	644.0	569.0	491.0	423.5		

G1	644.0	575.5	498.0	425.0	422.0
G2	644.0	577.0	505.5	433.5	423.0
G3	644.0	570.5	490.0	423.5	
G4	643.0	575.5	500.0	426.5	422.5
G5	644.0	577.5	502.0	428.5	420.5
C6	645.5	576.5	502.0	426.5	424.0
C7	645.0	573.5	499.0	427.0	424.5
C8	645.5	580.0	507.0	438.0	425.0
温度	25.8°C	25.4°C	25.7°C	26.1°C	
温度	31%	49%	45%	44%	

Platinum 7th floor

3h		4h		Downtime (All tanks No water remaining)
26.1ÿ/44%				
weight (g)	Spray volume (g)	weight (g)	Spray volume (g)	
421.0	61.5			171 min
428.0	74.0	422.0		190 min
431.0	72.0	421.5		198 min
423.5	70.5	422.0		182 min
422.0	68.5			176 min
434.5	70.0	422.0		198 min
422.5	63.5			171 min
429.0	74.0	423.5		190 min
423.5	67.5			176 min
425.0	73.0	422.0		186 min
433.5	72.0	423.0		192 min
423.5	66.5			174 min
426.5	73.5	422.5		186 min
428.5	73.5	420.5		193 min
426.5	75.5	424.0		184 min
427.0	72.0	424.5		186 min
438.0	69.0	425.0		195 min

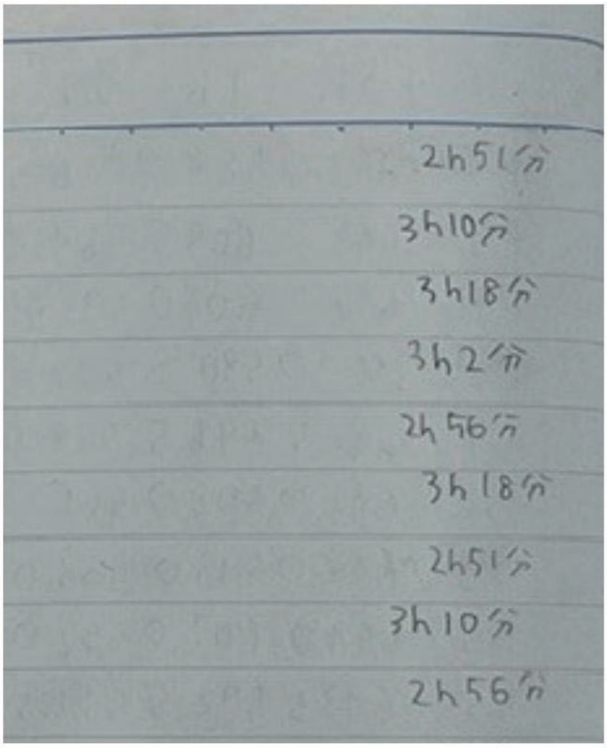

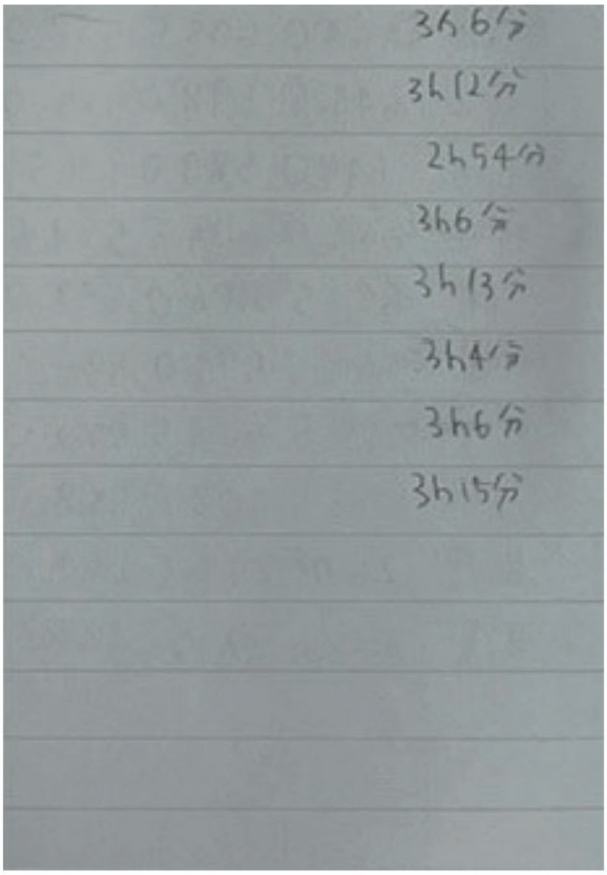
3h6分
3h12分
2h54分
3h6分
3h13分
3h4分
3h6分
3h15分

70mℓ/h＝1.17mℓ/min=1170μℓ/min＝19.5μℓ/sec
80 mℓ/h＝1.33mℓ/min=1330μℓ/min＝22.2μℓ/sec
